# Company Cost of Capital and Leverage: A Simplified Textbook Relationship Revisited

**DOI:** 10.1007/s41471-022-00144-w

**Published:** 2023-04-04

**Authors:** Valentin Haag, Christian Koziol

**Affiliations:** Department of Finance, University of Tübingen, Nauklerstraße 47, 72074 Tübingen, Germany

**Keywords:** Cost of capital, Default, Leverage, Firm value, Bankruptcy, G12, G32, G33

## Abstract

In this paper, we revisit a frequently employed simplification within the WACC approach that company cost of capital $k_{V}$ is supposed to be invariant to the debt ratio and therefore equal to the unlevered cost $k_{U}$. Even though we know from Miles and Ezzell (1980) that $k_{V}$ formally differs from $k_{U}$, treating both costs as equal strongly facilitates the practical firm valuation e.g. when companies strategically change their target debt ratios to a significantly different magnitude after a transaction. We provide both a theoretical model and an empirical analysis using 29 firms of the German stock market to quantify the economic significance between the company cost of a levered and an otherwise identical but unlevered firm. In particular, we can numerically support the usual simplification in the absence of default risk. In case that firms are default-risky, however, empirical findings indicate a clear difference between these costs equal to 1.88 percentage points on average even for moderate assumed bankruptcy costs which translates to a company mispricing of nearly 100%. As a result, the company cost of capital does practically not depend on the debt ratio if the firm is not subject to default risk or if bankruptcy costs are negligible. Otherwise, it does and a negligence of this relationship can cause significant mispricings.

## Introduction

For company valuation purposes, the weighted average cost of capital (WACC) approach is well-established and a frequently used tool. According to this discounted cash flow (DCF) method, the firm value results from discounting the company’s unlevered free cash flows net of taxes with the prominent WACC discount rate:

1 $$\text{WACC}=k_{V}-\frac{D}{V}\cdot k_{D}\cdot\tau=k_{E}\cdot\frac{E}{V}+k_{D}\cdot\frac{D}{V}\cdot\left(1-\tau\right)$$

Once the debt ratio $\frac{D}{V}$ (and correspondingly the equity ratio $\frac{E}{V}=1-\frac{D}{V}$), the company cost of capital $k_{V}$ (or alternatively the cost of equity $k_{E}$), the cost of debt $k_{D}$, and the corporate tax rate $\tau$ are known, we can easily determine the WACC in order to discount the unlevered free after tax cash flows of a levered company.

A typical case in business practice, however, is that a company might want to substantially change its debt ratio once such as e.g., after an acquisition, a private equity investment, or a leveraged buyout. Clearly, the new intended debt ratio $\frac{D}{V}$ should be taken into account for the present value of future cash flows so that the WACC discount rate needs to be adjusted, accordingly. While the direct effect from the debt ratio $\frac{D}{V}$ in Eq. (1) is trivial, the major issue still concerns the question whether the company cost of capital $k_{V}$ does change with the debt ratio $\frac{D}{V}$ in both an economic and a mathematical sense.

Even though the total cash flow approach (suggested by practically oriented financial auditors such as IDW (2014) on page 56) regards $k_{V}$ equal to the cost $k_{U}$ of the unlevered firm and therefore ignores any impact from the debt ratio $\frac{D}{V}$ on company cost of capital $k_{V}$, we find a mathematical effect when referring to some more sophisticated references. Already in Miles and Ezzell (1980) and accordingly in Sick (1990), we can find the following representation translated to our notation:

2 $$\begin{aligned}\text{WACC}=k_{U}-\tau\cdot k_{D}\cdot\frac{D}{V}\cdot\left(\frac{1+k_{U}}{1+k_{D}}\right)\end{aligned}$$

Equating both Eqs. (1) and (2), we obtain a simplified relation between $k_{V}$ and $k_{U}$:

3 $$\begin{aligned}k_{V}=k_{U}-\left(k_{U}-k_{D}\right)\cdot\frac{k_{D}}{1+k_{D}}\cdot\frac{D}{V}\cdot\tau\end{aligned}$$

We can easily see that there is a mathematical difference between $k_{V}$ and $k_{U}$ so that the debt ratio $\frac{D}{V}$ does impact $k_{V}$. Given a positive risk premium $k_{U}-k_{D}> 0$, the company cost of capital $k_{V}$ must be below the unlevered cost $k_{U}$. The numerical magnitude of this effect is, however, negligible. Even in an extreme case, with a risk premium $k_{U}-k_{D}$ equal to $8.00\%$, cost of debt $k_{D}$ equal to $2.00\%$, a debt ratio $\frac{D}{V}$ equal to $90\%$, and a tax rate equal to $35\%$, the company cost of capital $k_{V}$ for unlevered cost equal to $10.00\%$ amounts to $9.951\%$ and is nearly the same. This deviation of $0.049\%$ is usually far below the estimation accuracy of the cost of risky assets. Hence, this finding economically justifies the approach followed by IDW (2014) which does not regard any effect from the debt ratio on the cost $k_{V}$, on the one hand side, and still accounts for a marginal mathematical impact on the company cost of capital $k_{V}$ once the debt ratio changes, on the other side.

However, this practically helpful outcome bases on a model framework without any default risk. Therefore, the important question still remains whether the cost of capital $k_{V}$ might substantially change with the debt ratio $\frac{D}{V}$ if the firm is subject to default risk. Credit risk might be a crucial aspect for this relationship, because a higher debt ratio impacts the company in multiple ways such as the magnitude of the default probability, the amount of tax shields realized each period, and the triggering of additional losses due to default, also known as bankruptcy costs. For this reason, it is no longer obvious whether the company cost of capital $k_{V}$ is still sufficiently close to the unlevered cost $k_{U}$ for practical purposes and invariant of the debt ratio. Since the approximate equality between $k_{V}$ und $k_{U}$ is still frequently used in business practice, we need to investigate this relation in detail in order to justify or reject it for arbitrary cases.

The Covid-19 crisis has shown that the risk of a default is a highly relevant issue and additionally might change the established conditions. For example, under the German national bankruptcy regulations, the rules for filing for bankruptcy have been relaxed. Hence, our expectation is that the probability of default throughout the Covid-19 crisis was supposed to be reduced due to a postponed insolvency process. On the contrary, those companies, who had ultimately filed for bankruptcy, should have exhibited higher bankruptcy costs because of the additional time before formal bankruptcy in which those adverse effects took place.

It is astonishing, that there are only few recent developments of DCF valuation approaches that account for default risk. Kruschwitz et al. (2005) analyze the impact of a potential bankruptcy on the value of a firm under conditions of uncertainty. They deliberately assume identical gross cash flows, regardless of whether the company is more or less exposed to default risk and stress the difficulty concerning the quantification of the present value of bankruptcy costs. Cooper and Nyborg (2008) examine the impact of an investor’s personal taxes on the valuation of tax shields in the case of default-risky debt and compare their results with the adjustment formula of Miles and Ezzell (1980). Molnár and Nyborg (2013) expand this approach by the inclusion of possible recovery effects on the tax shields of a default-risky company. Koziol (2014) provides a closed-form solution for adjusting the WACC discount rate to account for default risk and bankruptcy costs. Koziol’s paper proposes a simple adjustment of the WACC rate to include both default risk and bankruptcy costs in a consistent firm valuation. Krause and Lahmann (2016) deal with the prioritization of principal or interest payments in the event of a default and evaluate the potential differences. Baule (2019) proposes a continuous-time model to illustrate the impact of default risk and bankruptcy costs on a firm’s cost of debt.

The aim of this paper is (i) to analyze the relation between company cost of capital $k_{V}$ and the debt ratio $\frac{D}{V}$ in the presence of default risk, (ii) to provide an economic understanding for its drivers, and (iii) to quantify potential pricing errors using empirical cases. When evaluating this question within the continuous-time framework by Leland (1994), we can confirm a relation similar to Eq. (3) that the company cost of capital $k_{V}$ is marginally below the unlevered cost $k_{U}$ unless default risk matters. However, the characteristics of $k_{V}$ dramatically change, once default risk is an issue for the firm. For those debt ratios, $k_{V}$ can strongly increase with $\frac{D}{V}$ and exceeds the cost of an unlevered firm excessively. After introducing a simple perodic DCF pricing model under default risk, we calibrate it to 29 companies from the German stock market to estimate the difference $k_{V}-k_{U}$ between the two different costs of capital and to evaluate its practical significance. Our empirical analysis reveals that the differences $k_{V}-k_{U}$ can be clearly depending on the size of bankruptcy costs. Even for moderate bankruptcy costs, we obtain a mean difference between $k_{V}$ and $k_{U}$ equal to 1.88 percentage points which creates a company mispricing of nearly 100%. In the special case of high bankruptcy costs resulting in a worthless firm after the default process, the average difference between $k_{V}$ and $k_{U}$ rises to 3.67 percentage points associated with arbitrarily increasing company mispricing errors. As a result, the company cost of capital $k_{V}$ does practically not depend on the debt ratio if the firm is not subject to default risk or if bankruptcy costs are negligible. Otherwise, for companies with economically significant default probabilities *and* bankruptcy costs, changes of the debt ratio strongly impact the company cost of capital and necessarily need to be considered.

The paper is structured as follows: In Sect. 2, we endogenously determine the company cost of capital in the prominent time-continuous model framework by Leland (1994) to demonstrate the fundamental effects of default risk and bankruptcy costs. In Sect. 3 we switch to a discrete time environment allowing for default risk and bankruptcy costs. Subsequently, we calibrate the theoretical model results with empirical data in Sect. 4. The paper concludes in Sect. 5. Technical developments are in the appendix.

## Endogenous company cost of capital within time-continuous Leland model

In this section, we regard a well-established model framework in continuous time, i.e. the Leland (1994) framework, in order to endogenously determine the company cost of capital for a firm subject to default risk. From Berk and DeMarzo (2014) on page 652 and Miles and Ezzell (1980) in Eq. (20), we can directly see that the company cost of capital does change when the firm changes its debt ratio. Still, as obtained from formula (3), the numerical differences between the company cost of capital $k_{V}$ and the cost $k_{U}$ of an otherwise identical but unlevered firm are negligible for typical parameter values. This observation justifies the usual practice to keep the company cost of capital $k_{V}$ constant even if the firm follows a change in its leverage policy.

However, this simple and fortunate conclusion must not necessarily hold for every particular firm. This is because Eq. (20) from Miles and Ezzell (1980) was explicitly derived for firms not exposed to default risk. In order to get an intuitive understanding how default risk impacts the relationship between company cost of capital $k_{V}$ and debt ratio and whether the practical rule that $k_{V}$ does not (considerably) change once the firms alters its debt ratio, we derive the instantaneous cost of capital within the Leland framework.

The Leland model considers a firm with a stochastic asset value $U$ that has the character of an unlevered firm value. Default risk comes from an outstanding debt obligation in form of a coupon stream $c$. Once the asset value $U$, that follows a geometric Brownian motion with standard deviation $\sigma$ of the return, hits the endogenous barrier $U_{B}$, the firm defaults. In this case, the firm is liquidated so that the debtholders obtain $(1-a)\cdot U_{B}$ taking proportionate bankruptcy costs $a$ into account and the equityholders are left with nothing. Otherwise, it is optimal for the firm/equity holders to pay the coupon which creates a tax shield equal to $\tau$ per unit of coupon paid. With the typical valuation assumptions and methods in a Black-Scholes world, the endogenous value $V\left(U\right)$ of a firm with unlevered firm value $U$ accounting for tax shields *and* bankruptcy costs results in

$$V\left(U\right)=U+\frac{c\cdot\tau}{r}+\left(\frac{U}{U_{B}}\right)^{-\frac{2r}{\sigma^{2}}}\cdot\left(-a\cdot U_{B}-\frac{c\cdot\tau}{r}\right)\,,$$

where $r$ stands for the risk-free rate and the default barrier amounts to $U_{B}=\frac{c\cdot\left(1-\tau\right)}{r+\frac{1}{2}\sigma^{2}}$. The firm value can be represented by a replicating portfolio consisting out of units of the unlevered firm $U$ and a risk-free asset. In Appendix I, we show that the dynamic, self-financing, replicating portfolio $RP$ exhibits the following portfolio weights $w_{U}$ and $w_{f}$ for holdings of the unlevered firm value $U$ and the risk-free asset, respectively:

$$\begin{aligned}\displaystyle&\displaystyle w_{U}=\frac{U+\frac{a\cdot U_{B}+\frac{c\cdot\tau}{r}}{\frac{\sigma^{2}}{2r}}\left(\frac{U}{U_{B}}\right)^{-\frac{2r}{\sigma^{2}}}}{V\left(U\right)}\\ \displaystyle&\displaystyle w_{f}=1-w_{U}.\end{aligned}$$

Since the company cost of capital coincides with the expected return obtained with the firm value, we endogenously determine the instantaneous return $\mu_{V}$ of the firm value (which has the character of the company cost of capital) for an instance of time. Once, the instantaneous return $\mu_{U}$ of the unlevered firm is given, the time-continuous company cost of capital $\mu_{V}$ reads:

$$\mu_{V}=\mu_{U}\cdot w_{U}+r\cdot w_{f}.$$

The debt ratio $\frac{D\left(U\right)}{V\left(U\right)}$ defined as the debt value over the firm value depends on the asset value $U$ and is given by:

$$\frac{D\left(U\right)}{V\left(U\right)}=\frac{\frac{c}{r}+\left(\frac{U}{U_{B}}\right)^{-\frac{2r}{\sigma^{2}}}\cdot\left(\left(1-a\right)\cdot U_{B}-\frac{c}{r}\right)}{U+\frac{c\cdot\tau}{r}+\left(\frac{U}{U_{B}}\right)^{-\frac{2r}{\sigma^{2}}}\cdot\left(-a\cdot U_{B}-\frac{c\cdot\tau}{r}\right)}$$

A crucial characteristic of the Leland model is that the asset value $U$ in relation to the default barrier $U_{B}$ determines the so called distance to default and therefore the default risk of the company as well as the debt ratio.

**Fig. 1:**
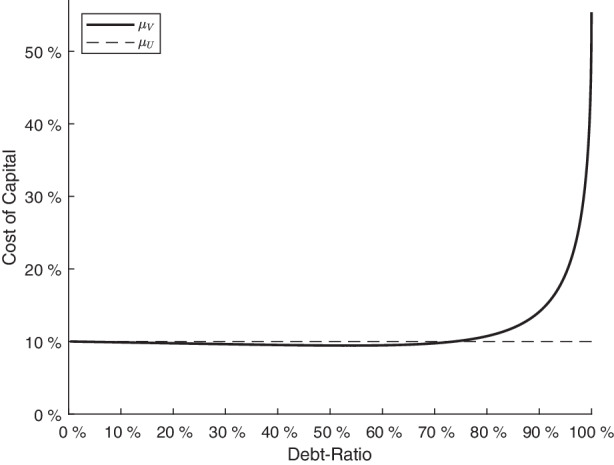
Company cost of capital $\mu_{V}$ for different debt ratios. *In this figure, endogenous cost of capital *$\mu_{V}$* is plotted for different debt ratios. The necessary input parameters are 0.10 for the instantaneous expected return *$\mu_{U}$*, 0.05 for the risk-free rate *$r$*, 1 for the coupon stream *$c$* of the outstanding debt obligation, 0.5 for the proportionate bankruptcy costs *$a$*, 0.25 for the tax rate *$\tau$* and 0.15 for the standard deviation *$\sigma$* of the return of the asset value U*

For $U\to U_{B}$, the firm is close to a default, the equity value is almost worthless and the debt ratio tends to one. On the other hand, for $U\to\infty$, the debt ratio approaches zero as the debt value cannot exceed its default-free value equal to $\frac{c}{r}$ and the firm value becomes arbitrarily large. Fig. 1 plots the company cost of capital $\mu_{V}$ for different debt ratios $\frac{D\left(U\right)}{V\left(U\right)}$. For debt ratios below 0.75, the firm exhibits a rather low default risk and therefore bankruptcy costs do not matter. In this region, the company cost of capital $\mu_{V}$ is (marginally) below the cost $\mu_{U}$ of an unlevered firm.

When the debt ratio, however, tends to one so that default risk as well as bankruptcy costs are an issue, the cost of capital $\mu_{V}$ increases heavily up to a limit equal to $55.37\%$. Formally, the limit can be written as:

4 $$\widehat{\mu}_{V}:=\underset{U\to U_{B}}{\lim}\mu_{V}=\mu_{U}+\left(\mu_{U}-r\right)\cdot\frac{a\cdot U_{B}+a\cdot U_{B}\frac{\sigma^{2}}{2r}+\frac{c\cdot\tau}{r}}{\left(1-a\right)\cdot U_{B}\frac{\sigma^{2}}{2r}}$$

The conclusion from this example in Fig. 1 is twofold: First, it confirms the findings from Berk and DeMarzo (2014) and Miles and Ezzell (1980) for the case without (or negligible default risk), i.e., debt ratios below 0.75. The intuition for why the company cost of capital $\mu_{V}$ is marginally below $\mu_{U}$ is because the levered firm can be represented by the unlevered firm and a risk-free position stemming from the (almost) risk-free tax shields. Thus, the levered firm is like a portfolio out of both positions (with positive holdings) so that the expected return, i.e., $\mu_{V}$, needs to be between the expected returns $\mu_{U}$ and $r$ of both particular positions. Due to the usual parameter values, the tax shields attribute to a minor part relative to the assets $U$, so that the company cost of capital $\mu_{V}$ is particularly close to the unlevered cost $\mu_{U}$.

Second, Fig. 1 also reveals that the company cost of capital is highly sensitive to changes in the debt ratio for default risky firms. The notion behind this important outcome from the Leland model is as follows: As a result of tax shields, which primarily matter in favorable states of the asset value $U$, and bankruptcy costs, which rise for low asset values $U$, the risk of the firm value $V\left(U\right)$ is higher than that of the unlevered firm value $U$. Illustratively speaking, when $U$ increases the firm value additionally benefits from tax shields, but a reduction of $U$ is additionally associated with a loss of the firm value $V\left(U\right)$ due to bankruptcy costs. Technically speaking, the replicating portfolio $RP=V\left(U\right)$ consists of a positive holding in the asset value $w_{U}\cdot RP> 0$ but the risk-free part of the replicating portfolio $w_{f}\cdot RP<0$ has a negative value (short position). Due to the prominent leverage effect, the expected return $\mu_{V}$ of the replicating portfolio, i.e., the company cost of capital, needs to exceed the expected return $\mu_{U}$ of the unlevered firm as long as the usual relation $\mu_{U}> r$ applies.

**Fig. 2:**
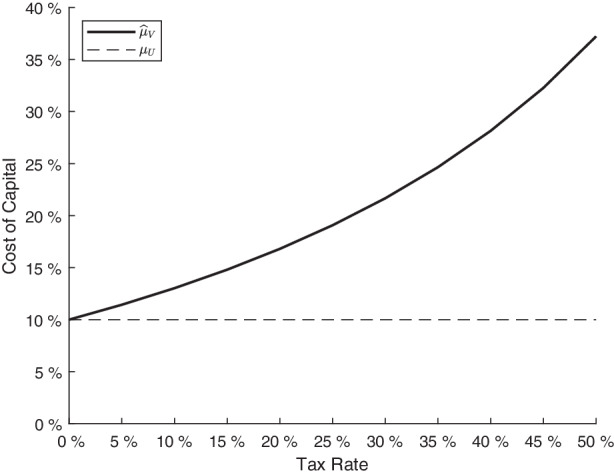
Maximum company cost of capital $\widehat{\mu}_{V}$ for different tax rates. *In this figure, the maximum cost of capital *$\widehat{\mu}_{V}$* is shown for different tax rates *$\tau$*. The necessary input parameters are 0.10 for the instantaneous expected return *$\mu_{U}$*, 0.05 for the risk-free rate *$r$*, 1 for the coupon stream *$c$* of the outstanding debt obligation, 0.15 for the standard deviation *$\sigma$* of the asset value *$U$* but no bankruptcy costs *$a$*. The debt ratio *$\frac{D}{V}$* for *$\widehat{\mu}_{V}$* amounts to 1 in all these cases, as the unlevered firm value *$U$* equals the lower barrier* $U_{B}$

A further important outcome from the company cost of capital $\mu_{V}$ in formula (4) with severe default risk is that bankruptcy costs play a major role for the value of $\mu_{V}$. When the relative bankruptcy costs $a$ increase to its maximum value one, the company cost of capital $\mu_{V}$ can become arbitrarily large. Still, even without bankruptcy costs, $a=0$, the company cost of capital $\mu_{V}$ can deviate substantially from $\mu_{U}$ due to tax shields. In this case for $a=0$, the limit simplifies to $\widehat{\mu}_{V}=\mu_{U}+\left(\mu_{U}-r\right)\cdot\frac{2r+\sigma^{2}}{\sigma^{2}}\frac{\tau}{1-\tau}$. In line with Fig. 2, the limit $\widehat{\mu}_{V}$ of the company cost of capital increases with the tax rate. While in the absence of taxes $\tau=0$, $\widehat{\mu}_{V}$ coincides with $\mu_{U}=10\%$, it is fundamentally higher for typical tax rates. For $\tau=25\%$, the limit $\widehat{\mu}_{V}$ amounts to $19.1\%$ and it even obtains a value equal to $37.2\%$ when the tax rate is $50\%$.

## Company cost of capital in a Discrete Time Environment

### Unlevered Firm

In order to have an environment that can be calibrated to real levered companies, we no longer stick to the continuous version but switch to a discrete time environment. This allows us, to determine the cost of capital of a levered company under impact of default risk and bankruptcy costs. For this purpose, we specify a tractable framework with time-invariant characteristics and infinite lifetime.

We first regard the valuation according to discounted cash flows (DCF) of a fictitious unlevered firm, then we relate it to a levered firm.^1^ In particular, we impose the following six assumptions:

1. The firm generates positive free cash flows $X_{t}$ after tax at each discrete date $t$. Strictly speaking, the cash flows $X_{t}$ are those of an otherwise identical but unlevered firm. Consequently, the tax shield is not taken into account within $X_{t}$, regardless of the amount of debt.
2. The firm operates on a perfect market free of arbitrage opportunities, perfect competition and without capital frictions except for corporate taxes. Personal taxes on investor basis are ignored for simplicity.
3. The valuation of all corporate claims is related to the value $U_{t}$ of the unlevered firm and on the risk-free asset. Both, the cost of capital $k_{U}$ of the unlevered company and the risk-free rate $r_{f}$ are constant for all future periods. Alternatively, $k_{U}$ can be understood as the expected return of the unlevered firm or the risk-adjusted discount rate for the expected revenues from an investment into the unlevered firm. Due to a positive systematic risk, we consider cost $k_{U}$ of an unlevered firm to be above $r_{f}$.
4. The firm exists infinitely long with discrete time steps $t$ at all (integer) dates.
5. The firm can adopt one of two different states in each period. In the first state (the up-state), the firm exhibits an conditional expected gross cash flow growth rate of $u=\frac{\mathbb{E}_{t}\left(X_{t+1}\mid\text{up-state}\right)}{X_{t}}$. In the second state, the firm has an conditional gross cash flow growth rate of $d=\frac{\mathbb{E}_{t}\left(X_{t+1}\mid\text{down-state}\right)}{X_{t}}$. The respective states of the firm occur with probabilities $1-p$ (up-state) and $p$ (down-state). The conditional expected growth rate of the firm amounts to $g=\left(1-p\right)\cdot u+p\cdot d-1$. We note that the down-state reflects one feasible cash flow $X_{t+1}$ at which the illiquidity condition must be satisfied, while the up-state can comprise of arbitrarily many different cash flow levels $X_{t+1}$ with conditional mean $\mathbb{E}_{t}\left(X_{t+1}\mid\text{up-state}\right)$.^2^
6. As long as the firm remains unlevered, i.e. completely financed by equity, it is not subject to default risk and bankruptcy costs.^3^

**Fig. 3:**
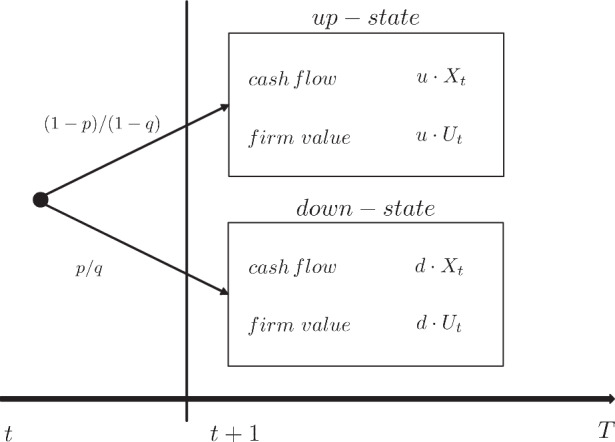
Binomial tree of an unlevered firm

In order to determine the market value of the unlevered firm $U_{t}$, we first refer to risk-neutral probabilities $1-q$ and $q$. The existence of such probabilities directly follows from the assumption of no-arbitrage (assumption 2). When using the risk-neutral probabilities $1-q$ and $q$ rather than the true probabilities $1-p$ and $p$, the expected cash flows must be discounted at the risk-free rate $r_{f}$ to obtain the value of the corresponding claim. In line with assumption 3, we regard a relationship from time $t$ to $t+1$ based on the binomial tree described for the otherwise identical but unlevered firm with given company cost of capital $k_{U}$. We can describe the firm value $U_{t}$ in two ways: First, in relation to the true probabilities $1-p$ and $p$ and second in relation to the risk-neutral probabilities $1-q$ and $q$ as shown in Fig. 3. As a consequence of the fact that an unlevered firm is never subject to default risk, the proceeds of an investment into the firm at time $t=1$ comprise of the free cash flow $X_{t+1}$ and the unlevered firm value $U_{t+1}$:

5 $$\begin{aligned}U_{t}=\frac{\left(1-p\right)\cdot(u\cdot X_{t}+u\cdot U_{t})+p\cdot(d\cdot X_{t}+d\cdot U_{t})}{1+k_{U}}\end{aligned}$$

In the case of the up-state, the expected free cash flow and the expected firm value increase by factor $u$ with probability $1-p$. Conversely, in the down-state, the free cash flow and the firm value decrease by factor *d* with probability $p$. The reason why the unlevered firm value $U_{t}$ is multiplied by $u$ in the up-state and by $d$ in the down-state directly follows from the first degree homogeneity of $U_{t}$ in $X_{t}$.^4^ Related to the special case in the binomial tree, such a multiplier $f_{U}$ with $U_{t}=f_{U}\cdot X_{t}$ exists for any point in time $t$. Since the firm value $U_{t}$ must also coincide with the corresponding representation according to risk-neutral valuation obtain the following formula using the risk-neutral probability $q$ and discounting by $r_{f}$:

6 $$\begin{aligned}U_{t}=\frac{\left(1-q\right)\cdot(u\cdot X_{t}+u\cdot U_{t})+q\cdot(d\cdot X_{t}+d\cdot U_{t})}{1+r_{f}}\end{aligned}$$

Solving Eq. (6) for $U_{t}$ provides us with:

7 $$\begin{aligned}U_{t}=\frac{(1-q)\cdot u+q\cdot d}{1+r_{f}-(1-q)\cdot u-q\cdot d}\cdot X_{t}=f_{U}\cdot X_{t}\end{aligned}$$

As we can see in Eq. (7), the endogoneous scaling factor $f_{U}$ for unlevered firm value $U_{t}$ amounts to

$$f_{U}=\frac{(1-q)\cdot u+q\cdot d}{1+r_{f}-(1-q)\cdot u-q\cdot d}.$$

Conversely, taking into account the real probabilities $p$ and $1-p$, the following relationship is obtained for $f_{U}$:

8 $$\begin{aligned}f_{U}=\frac{(1-p)\cdot u+p\cdot d}{1+k_{U}-(1-p)\cdot u-p\cdot d}\end{aligned}$$

Since $g=\left(1-p\right)\cdot u+p\cdot d-1$ holds with reference to assumption 5, we can simplify $f_{U}$ as follows:

$$\begin{aligned}\displaystyle f_{U}=\frac{1+g}{k_{U}-g}\end{aligned}$$

If we now combine Eqs. (5) and (6) with each other, we obtain the following formula for a solution for the risk-neutral probability $q$:

9 $$\begin{aligned}q=1-\frac{1}{1+k_{U}}\cdot\left(\left(1-p\right)\cdot\left(1+r_{f}\right)-\frac{d\cdot\left(k_{U}-r_{f}\right)}{u-d}\right)\end{aligned}$$

These probabilities are helpful to price the otherwise identical but levered firm value claims. Since $k_{U}> r_{f}$ applies according to assumption 3, $q> p$ must therefore be valid. In other words, the greater the difference between the cost of capital $k_{U}$ and $r_{f}$, the greater the difference between the risk-neutral probability $q$ and the true down-state probability $p$ holding all other factors fixed. Note that the risk-neutral probability $1-q$ must range between 0 and 1 to ensure the no arbitrage condition. Hence, the condition $1+r_{f}> \left(1-q\right)\cdot u+q\cdot d$ from the denominator of Eq. (7) must be met, which also ensures solely finite values for the firm value $U_{t}$.

### Levered Firm

The ability to represent the risk-neutral probability $q$ as a function of $p$ allows us to determine the corresponding market value of a levered firm $V_{t}$ taking debt financing into account. Once the market value of the levered firm $V_{t}$ is known, the cost of capital of the levered firm $k_{V}$ as well as the cost of equity $k_{E}$ and the cost of debt $k_{D}$ can be derived. For this, additional assumptions regarding the firm’s financing policy are necessary, while the assumptions already presented for the company without debt remain valid:

7. All residual cash flows are distributed to the equity holders after remunerating the debt holders.
8. The firm always issues one-periodical debt. If debt capital in the amount of $D_{t}$ is issued at time $t$, this contract must be redeemed at time $t+1$ with $D_{t}\cdot(1+c)$. The interest rate $c$ is the required rate to issue debt for one period at a consistent market value $D_{t}$.
9. In the event of a default, bankruptcy costs occur. In order to incorporate proportionate bankruptcy costs $\alpha$ into our model framework, we relate them to the respective previous period. Thus, if the firm defaults at time $t+1$, we calculate the bankruptcy costs in relation to the firm value $V_{t}$ of the previous period $t$ (see for example Koziol 2014). The lower residual value of the firm after bankruptcy costs is for the debt holders while the equity holders are left with nothing. Obviously, the residual value $d\cdot X_{t}+d\cdot V_{t}-\alpha\cdot V_{t}$ must be less than interest and repayments, i.e. $c\cdot D_{t}+D_{t}$ to satisfy the default condition. Since the choice of bankruptcy costs $\alpha$ is flexible, we can control the payments and/or values in the default event, e.g., for different points in time and/or partial interest payments and interpret the values and/or payoffs are the outcome of both overindebtedness and inability to pay.
10. Tax shields occur due to tax-deductible cost of debt financing as long as the firm is solvent. We assume a constant tax rate $\tau$ over the firm’s lifetime $T$. If the company defaults, the company is also unable to pay its interest on debt capital. Accordingly, the tax benefits in the state of default disappear.
11. The firm proposes a market value oriented financing policy so that the debt-ratio $\frac{D}{V}$ is constant over time.^5^ Based on the initial assumption that the debt volume is proportional to the cash flows $D_{t}=f_{D}\cdot X_{t}$, we can show that $c$ is constant due to constant discount rates $k_{U}$ and $r_{f}$. These properties ultimately result in a constant debt ratio $\frac{D}{V}$. We provide the proof hereof in Appendix II.

The ability to represent the risk-neutral down-state probability $q$ as a function of $p$ allows us to determine the market value of $D_{t}$ and $E_{t}$ under the inclusion of debt financing as a second step. For this purpose, we use the considered binomial model and relate it to both equity and debt payoffs at dates $t+1$ and $t$. Since the firm is now levered, the down-state probability $q$ means the default of the firm.

The debt holders invest capital in the amount of $D_{t}$ at time $t$. If the firm does not default at time $t+1$, the debt holders receive the interest $c\cdot D_{t}$ as well as the redemption of the invested capital $D_{t}$, which equals $D_{t}\cdot\left(1+c\right)$ in total. If the firm defaults at $t+1$ (down-state), the debt holders receive the full residual value of the company. This value comprises of the cash flow of the company $d\cdot X_{t}$ and the firm value $d\cdot V_{t}$ net of bankruptcy costs $\alpha\cdot V_{t}$. Therefore, we obtain from risk-neutral valuation:

10 $$\begin{aligned}D_{t}=(1-q)\cdot\frac{D_{t}\cdot\left(1+c\right)}{1+r_{f}}+q\cdot\frac{d\cdot X_{t}+\left(d-\alpha\right)\cdot V_{t}}{1+r_{f}}\end{aligned}$$

The equity holders receive the cash flow $u\cdot X_{t}$ as well as the tax benefits $D_{t}\cdot c\cdot\tau$ from the debt issuance, if the firm does not default at time $t+1$. However, the interest of the debt holders $c\cdot D_{t}$ still has to be paid by the equity holders. Since new one-periodical debt $u\cdot D_{t}$ is issued at time $t+1$ and the existing debt $D_{t}$ is redeemed, the net issuance proceeds $u\cdot D_{t}-D_{t}$ are also in favor of the equity holders. The last position is the equity value $E_{t+1}$ equal to $u\cdot E_{t}$. If the firm defaults at time $t+1$, the equity holders are left with nothing. Therefore, the equity value $E_{t}$ can be represented by risk-neutral valuation as follows:

11 $$\begin{aligned}E_{t}=(1-q)\cdot\frac{u\cdot X_{t}+D_{t}\cdot c\cdot\tau-D_{t}\cdot c+u\cdot D_{t}-D_{t}+u\cdot E_{t}}{1+r_{f}}\end{aligned}$$

**Fig. 4:**
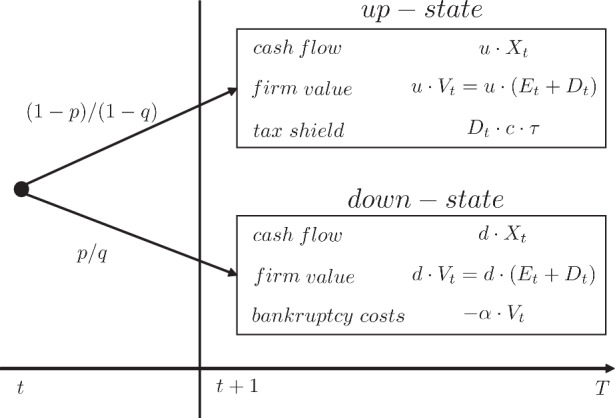
Binomial tree of a levered firm

If we summarize the levered firm $V_{t}$ as a portfolio of equity and debt, several transfer payments between equity and debt holders are eliminated. The residual payoffs of the levered firm $V_{t}$ are illustrated in Fig. 4 accounting for both the tax advantages as well as the disadvantage from debt, i.e., default risk and the corresponding bankruptcy costs.

In order to derive the market value for $V_{t}$ at time $t$, we can write according to risk-neutral valuation:

12 $$\begin{aligned}V_{t}=\frac{(1-q)\cdot(u\cdot X_{t}+u\cdot V_{t}+D_{t}\cdot c\cdot\tau)+q\cdot(d\cdot X_{t}+d\cdot V_{t}-\alpha\cdot V_{t})}{1+r_{f}}\end{aligned}$$

For a constant given debt ratio $\frac{D}{V}$ in line with assumption 10 we can write $D_{t}=\frac{D}{V}\cdot V_{t}$ and solve Eq. (12) for $V_{t}$:

13 $$\begin{aligned}V_{t}=\frac{(1-q)\cdot u\cdot X_{t}+q\cdot d\cdot X_{t}}{(1+r_{f})-(1-q)\cdot(u+\frac{D}{V}\cdot c\cdot\tau)-q\cdot(d-\alpha)}\end{aligned}$$

Since the debt interest rate $c$ is an endogenous variable within the model with default risk, we can derive $c$ from the market value of the debt capital $D_{t}$. If we insert Eq. (13) into Eq. (10), we obtain the following solution for $c$:

14 $$\begin{aligned}c=\frac{\frac{D}{V}\cdot\left(q+r_{f}\right)\cdot\left(d\cdot q+\left(1-q\right)\cdot u\right)-q\cdot\left(d\cdot\left(1+r_{f}\right)-\left(1-q\right)\cdot u\cdot\alpha\right)}{\frac{D}{V}\cdot\left(1-q\right)\cdot\left(d\cdot q\cdot\left(1-\tau\right)+\left(1-q\right)\cdot u\right)}\end{aligned}$$

Likewise, we can also insert Eq. (14) into Eq. (13) to finally obtain the following term for $V_{t}$ irrespective of the interest rate $c$:

15 $$\begin{aligned}V_{t}=\frac{\left(1-q\right)\cdot u\cdot X_{t}+q\cdot d\cdot X_{t}\cdot\left(1-\tau\right)}{1+r_{f}-\left(1-q\right)\cdot u-q\cdot\left(d-\alpha\cdot\left(1-\tau\right)-d\cdot\tau+\tau\cdot\frac{D}{V}\right)-r_{f}\cdot\tau\cdot\frac{D}{V}}\end{aligned}$$

Since the cost of capital is equivalent to the expected return from an one-period investment, we can now compute the endogenous value of the cost of capital $k_{V}$. For this purpose, we simply have to relate the total expected payoffs using the true probability $p$ obtained from an investment at time $t+1$ to the invested capital into the corresponding claim:

16 $$\begin{aligned}k_{V}=\frac{(1-p)\cdot(u\cdot X_{t}+u\cdot V_{t}+D_{t}\cdot c\cdot\tau)+p\cdot(d\cdot X_{t}+d\cdot V_{t}-\alpha\cdot V_{t})}{V_{t}}-1\end{aligned}$$

The cost of capital $k_{V}$ in Eq. (16) is shown as a function of the amount of unlevered cash flows $X_{t}$. Since the value of the levered firm $V_{t}$ is homogenous of degree one in $X_{t}$ (proof in Appendix II) we can calculate the cost of capital $k_{V}$ using the multiplier $f_{V}$ implicitly defined by $V_{t}=f_{V}\cdot X_{t}$:

17 $$\begin{aligned}k_{V}=\frac{\left(1-p\right)\cdot\left(u+u\cdot f_{V}+f_{V}\cdot\frac{D}{V}\cdot c\cdot\tau\right)+p\cdot\left(d+d\cdot f_{V}-\alpha\cdot f_{V}\right)}{f_{V}}-1\end{aligned}$$

with:

18 $$\begin{aligned}f_{V}=\frac{\left(1-q\right)\cdot u+q\cdot d\cdot\left(1-\tau\right)}{1+r_{f}-\left(1-q\right)\cdot u-q\cdot\left(d-\alpha\cdot\left(1-\tau\right)-d\cdot\tau+\tau\cdot\frac{D}{V}\right)-r_{f}\cdot\tau\cdot\frac{D}{V}}\end{aligned}$$

Based on an one-periodical investment into the market values of debt $D_{t}$ and equity $E_{t}$ specified by Eqs. (10) and (11), the cost of debt capital $k_{D}$ and the cost of equity $k_{E}$ can be written analogously to the derivation of the cost of capital $k_{V}$ using the true default probability $p$:

19 $$\begin{aligned}k_{D}=\frac{\left(1-p\right)\cdot D_{t}\cdot\left(1+c\right)+p\cdot\left(d\cdot X_{t}+\left(d-\alpha\right)\cdot V_{t}\right)}{D_{t}}-1\end{aligned}$$

20 $$\begin{aligned}k_{E}=\frac{(1-p)\cdot\left(u\cdot X_{t}+u\cdot E_{t}+\left(u-1\right)\cdot D_{t}-D_{t}\cdot c\cdot\left(1-\tau\right)\right)}{E_{t}}-1\end{aligned}$$

In order to further evaluate the impact of bankruptcy costs on the firm value in Eq. (15), we regard the feasible range for the bankruptcy costs $\alpha$. Since bankruptcy costs are borne entirely by the debt holders, we define $\hat{\alpha}$ as maximum value for the bankruptcy costs with a non-negative residual value in the event of bankruptcy. Since the value of a firm in the event of default is $d\cdot X_{t}+\left(d-\alpha\right)\cdot V_{t}$, we can determine $\hat{\alpha}$ with the following relationship:

$$\begin{aligned}\displaystyle d\cdot X_{t}+\left(d-\hat{\alpha}\right)\cdot V_{t}\overset{!}{=}0\end{aligned}$$

If we use Eq. (15) for the firm value $V_{t}$ and solve for $\hat{\alpha}$, we get the maximum feasible value for the bankruptcy costs $\alpha$:

$$\begin{aligned}\displaystyle\hat{\alpha}=\frac{d\cdot\left(\left(q+r_{f}\right)\cdot\tau\cdot\frac{D}{V}-1-r_{f}\right)}{u\cdot\left(q-1\right)}\end{aligned}$$

We exemplarily compute the company cost of capital $k_{V}$ in the presence of default risk as a function of the relative bankruptcy costs $\alpha$.

**Table 1:** Difference between $k_{V}$ and $k_{U}$ with increasing bankruptcy costs. *In this table, we illustrate the pricing error PE for different levels of the bankruptcy costs *$\alpha$*. The necessary input parameters are 0.10 for the unlevered cost of capital *$k_{U}$*, 0.05 for the risk-free rate *$r_{f}$*, 0.6 for the debt ratio *$\frac{D}{V}$*, 0.01 for the one-period default probability *$p$*, 1.09 for the up-state factor *$u$* and 0.6 for the down-state factor *$d$*. The tax rate *$\tau$* amounts to 0.30. These parameter values result in a risk-neutral probability *$q$* equal to 0.11. The maximum bankruptcy costs *$\hat{\alpha}$* of this exemplary firm is 0.632*

$\alpha$	$c$	$k_{V}$	$k_{V}-k_{U}$	PE
0%	5.56%	10.06%	0.06%	3.9%
5%	6.55%	10.60%	0.60%	40.2%
10%	7.54%	11.14%	1.14%	76.4%
15%	8.53%	11.68%	1.68%	112.6%
20%	9.52%	12.22%	2.22%	148.9%
25%	10.51%	12.76%	2.76%	185.1%
30%	11.49%	13.30%	3.30%	221.4%
35%	12.48%	13.84%	3.84%	257.6%
40%	13.47%	14.38%	4.38%	293.9%
45%	14.46%	14.92%	4.92%	330.1%
50%	15.45%	15.46%	5.46%	366.3%
55%	16.44%	16.00%	6.00%	402.6%
60%	17.43%	16.54%	6.54%	438.8%
$\hat{\alpha}=63.2\%$	18.06%	16.88%	6.88%	462.0%

Table 1 shows the endogenous values of the interest rate $c$ as well as the company cost of capital $k_{V}$ of a levered firm. The cost of capital $k_{V}$ increases when the bankruptcy costs rise. As we can see from Table 1, even for zero bankruptcy costs, $k_{V}$ is still higher than $k_{U}$. The intuition for this relationship is because the return of a levered firm in the favorable state is higher than that of an unlevered firm due to tax shields but lower than that of an unlevered firm in the unfavorable state due to bankruptcy costs. Hence, we can replicate the levered firm by a portfolio out of holdings of the unlevered firm and a risk-free credit. This view is similar to the representation within the Leland framework carried out in Sect. 2. As a result of a positive risk premium $k_{U}> r_{f}$, the levered firm must have an expected return (i.e., cost of capital) above $k_{U}$ due to the leverage effect. The formal proof that the cost of capital $k_{V}$ of a levered company exceeds the cost of capital $k_{U}$ of a company without debt even in the case of $\alpha=0$ is provided in Appendix III.

To illustrate the magnitude of the pricing error of a wrong discount rate, we consider a perpetual expected cash flow stream $X_{t}$ with expected growth $g$. The pricing error demonstrated in the last column of Table 1 is the percentage difference between the values for the “correct” discount rate $k$ relative to a wrong one $k^{\prime}$. In other words, if $\frac{X_{t}}{k-g}$ is the “true” value and $\frac{X_{t}}{k^{\prime}-g}$ is the “wrong” value, the percentage difference is

21 $$\begin{aligned}\text{PE} & =\frac{\frac{X_{t}}{k^{\prime}-g}}{\frac{X_{t}}{k-g}}-1=\frac{k-g}{k^{\prime}-g}-1=\frac{k-k^{\prime}}{k^{\prime}-g}.\end{aligned}$$

In the present case the “correct” discount rate $k$ equals $k_{V}$ and the “wrong” discount rate $k^{\prime}$ equates $k_{U}$. The growth rate $g$ is $\left(1-p\right)\cdot u+p\cdot d-1$.

Even for relatively low bankruptcy costs of 20%, we find tremendous deviations. In the present case, the discount rates deviate by 2.22%, resulting in a pricing error of 148.9%. In fact, $k_{V}$ exhibits a significantly higher value than $k_{U}$, in contrast to the adjustment according to Miles and Ezzell (1980), where $k_{U}$ is even slightly higher than $k_{V}$. Thus, if a firm was evaluated according to the practicable simplification $k_{V}=k_{U}$, the value of the firm would be overestimated by nearly two and a half times.

If companies are fully liquidated in the event of a default or if a company loses key players such as managers or scientists after going bankrupt, bankruptcy costs at or close to $\hat{\alpha}$ are feasible. Hence, pricing errors can easily rise to 462% in this example for bankruptcy costs converging to $\hat{\alpha}$.

Table 2 illustrates the impact of default risk while the bankruptcy costs $\alpha$ remain constant at 40%. We start at a 1-period probability of default of 0.5%, which corresponds to a rating of AAA–AA, and increase in ten steps to 5.5%, which corresponds to a rating of B$-$ to C.^6^ It is surprising that a variation in the magnitude of the probability of default barely affects the difference between $k_{V}$ and $k_{U}$, while the pricing error PE rises strongly. At first glance, this is not intuitive, because with a decreasing probability of default one would expect a lower effect of the bankruptcy costs. As an opposite effect, the growth rate $g$ must be considered here: Since the state factors $u=1.09$ and $d=0.60$ are fixed in this example, the impact of $u$ increases with a decreasing probability of default $p$. Therefore, the growth factor $g$ in Eq. (21) declines with increasing default risk $p$ so that the Pricing Error PE rises.

**Table 2:** Difference between $k_{V}$ and $k_{U}$ with increasing default risk. *In this table, we illustrate the pricing error PE for different levels of default risk *$p$*. The necessary input parameters are 0.10 for the unlevered cost of capital *$k_{U}$*, 0.05 for the risk-free rate *$r_{f}$*, 0.6 for the debt ratio *$\frac{D}{V}$*, 1.09 for the up-state factor *$u$* and 0.6 for the down-state factor *$d$*. The tax rate *$\tau$* is 0.30 and the bankruptcy costs *$\alpha$* amount to 0.40*

$p$	$q$	$c$	$g$	$k_{V}$	$k_{V}-k_{U}$	*PE*
5.5%	0.154	16.73%	6.31%	14.41%	4.41%	119.4%
5.0%	0.149	16.37%	6.55%	14.41%	4.41%	127.8%
4.5%	0.144	16.01%	6.80%	14.41%	4.41%	137.4%
4.0%	0.139	15.65%	7.04%	14.40%	4.40%	148.7%
3.5%	0.135	15.29%	7.28%	14.40%	4.40%	162.0%
3.0%	0.130	14.93%	7.53%	14.39%	4.39%	177.9%
2.5%	0.125	14.57%	7.78%	14.39%	4.39%	197.3%
2.0%	0.120	14.20%	8.02%	14.39%	4.39%	221.5%
1.5%	0.115	13.84%	8.27%	14.38%	4.38%	252.6%
1.0%	0.111	13.47%	8.51%	14.38%	4.38%	293.9%
0.5%	0.106	13.11%	8.76%	14.37%	4.37%	351.4%

In a nutshell, we can state that the cost of capital $k_{V}$ of a levered firm exceeds the cost of capital $k_{U}$ of an unlevered firm as soon as default risk plays a role. In particular, the level of the bankruptcy costs $\alpha$ exhibits a strong impact on the capital cost $k_{V}$ of the levered firm. In addition, the simplifying assumption $k_{V}=k_{U}$ may no longer be justified in this case, since this effect is of a remarkable economical significance with serious pricing errors especially for firms with relatively high bankruptcy costs.

## Model Calibration

In this chapter, we document the economic significance of the difference between $k_{V}$ and $k_{U}$ using real firms. The intention of this chapter is not to provide general results for companies with different levels of debt or default risk. This would be nearly impossible due to the heterogeneity of companies in terms of financing policy, industry or operating activities. Rather, the objective is to show that real companies exhibit economically significant differences in $k_{V}$ as soon as bankruptcy costs are considered.

Therefore, we will firstly demonstrate the calibration of the model using an example of the US capital market with severe default risk, and then secondly apply to a larger data set to demonstrate that each firm can be valued within the existing model framework.

With „Range Resources Corporation“, we first choose a company in the US American oil and gas production sector that exploits conventional energy sources, particularly by using „fracking“ technology in the USA. Especially, the drop in oil prices in the years 2014–2017 had a negative impact on the credit rating of Range Resources. With Caa1 (Moody’s) and BB$+$ (StandardPoors) the company exhibits rating at non-investment grade. Further, we choose January 1, 2018 as the valuation date. To calibrate the model analogous to the theoretical considerations in Chapt. 3, the following exogenous parameters are essential:

**Table 3:** Exogenous Parameters of Range Resources Corp.

$r_{f}$	$\frac{D}{V}$	$u$	$\tau$	$p$	$k_{E}$	$c$
2.82%	58.45%	1.02	35%	5.37%	7.62%	5.79%

The risk-free interest rate $r_{f}$ is derived from the yields of 30-year US American government bonds and amounts to 2.82% as at January 1, 2018. The Company reports total liabilities of USD 5,954.6 million at December 31, 2017. The market capitalization amounts to USD 4,233.3 million, resulting in a debt ratio $\frac{D}{V}$ of 58.4%, which we assume to be constant for the future. The US corporate tax rate of 35% is supposed to be constant as well. We also assume that the company will continue at a moderate growth rate of $u=1.02$ in the case of solvency.

There are different approaches and models in financial literature to estimate the probability of default of firms with the help of scoring and rating models. Still, a simple and objective way to derive a firm’s probability of default $p$ is to refer to external rating data of the firm and to use these rating scores to derive the probability of default from historical default frequencies of firms from the corresponding rating class. To get a value as stable as possible for the one-year probability of default $pd_{1}$, we use the 10-year default probability $pd_{10}$ and convert this into the corresponding one-year probability of default $pd_{1}$ as exemplarly shown at Hartmann-Wendels et al. (2019), page 439:

$$\begin{aligned}\displaystyle pd_{1}=1-\left(1-pd_{10}\right)^{\frac{1}{10}}\end{aligned}$$

The rating grades from Moody’s and StandardPoors mentioned at the beginning of this section result in a weighted one-period probability of default of 5.37%.

Concerning the bankruptcy costs $\alpha$, practical examples show that in case of a post-bankruptcy liquidation, up to 100% of the assets of a firm before bankruptcy can be completely lost. In scientific discourse a distinction is made between direct and indirect bankruptcy costs, which affect companies in total in the event of a default. Direct bankruptcy costs comprise legal expenses, court costs or advisory fees. The indirect bankruptcy costs are apparent in the loss of reputation, important customers and employees as well as the potential loss due to a fire sale of assets or an inefficient liquidation process. Various empirical studies concerning direct bankruptcy costs have been published, for example Baxter (1967); Warner (1977); Altman (1984); Weiss (1990); Betker (1997); Lubben (2000); Thorburn (2000) and LoPucki and Doherty (2004). These studies show average values between 2 and 7% for large companies, depending on the sample examined. Most studies only publish mean values, whereas the outer maximum percentiles are also of major interest. Betker (1997) mentions here, for example, a maximum value of 14%. The values also depend very strongly on the size of the firms within the sample. In the case of Chapt. 7 liquidations, Lawless and Ferris (1997) report a maximum loss of 96.1% close to a total loss of assets due to bankruptcy. This case corresponds to bankruptcy costs equal to $\hat{\alpha}$ in our framework. Indirect bankruptcy costs are typically higher than direct bankruptcy costs and more difficult to quantify accurately. Therefore, the number of empirical studies is also smaller, among which, for example, Kwansa and Cho (1995); Andrade and Kaplan (1998) and Bhabra and Yao (2011) should be mentioned. Bhabra and Yao (2011) differentiate between different sectors and show relatively high indirect bankruptcy costs for very research-intensive and personnel-dependent technology firms, averaging up to 27%. Kwansa and Cho (1995) document maximum values of 43.2%. In total, i.e. direct and indirect bankruptcy costs combined, large firms across all sectors can exhibit severe magnitudes and very strongly among sectors. Due to the fact that up to 100% of the assets can be lost in the event of a liquidation following bankruptcy, we present the full range of bankruptcy costs in this model calibration.

Both, the cost of capital $k_{U}$ of fictitiously unlevered firms and the down factor $d$ are not intuitively observable on the capital market. Thus, we consider two proxies, $k_{E}$ and $c$, to calibrate $k_{U}$ and $d$ by using two conditions and Eq. (9). In particular, we apply the interest rate $c$ as calibration constraint (A) and the cost of equity $k_{E}$ as calibration constraint (B) to simultaneously estimate $k_{U}$ and $d$.

For the calibrations constraint (A), we empirically estimate the interest rate $\hat{c}$ using credit spreads from bonds issued by Range Resources. Since in the present case a total of 13 bonds with different maturities were issued on the valuation date, we use these bonds to determine a maturity-weighted credit spread and thus determine the company’s interest rate on debt. This procedure results in an empirical observed interest rate $\hat{c}$ of 5.79%.

In order to use $k_{E}$ within the framework of calibration constraint (B) for $k_{U}$, we determine the estimate $\hat{k}_{E}$ using the well-known capital asset pricing model and use the firm’s beta factor $\beta_{E}$ and the market risk premium $\mu_{\text{BM}}-r_{f}$ in the following well-known way:

$$\begin{aligned}\displaystyle\hat{k}_{E}=r_{f}+\beta_{E}\cdot\left(\mu_{\text{BM}}-r_{f}\right)\end{aligned}$$

We calculate the beta factor $\beta_{E}$ using historical share prices for the three years 2015, 2016, and 2017 and regress daily returns of the single shares against the daily returns of the broadly diversified SP 500 index, which amounts to 1.17. Subsequently, we calculate the market return $\mu_{\text{BM}}$ using the historical average of 1‑year returns of the MSCI World from 2000-2017 resulting in a market return of 6.92% and a market risk premium of 4.10%. Thus, the empirically measured cost of equity $\hat{k}_{E}$ amounts to 7.62%.

To relate the empirical estimate $\hat{k}_{E}$ to the endogenous outcome $k_{E}$ from our model, we can use Eq. (20) for the cost of equity $k_{E}$ as expected equity return. From the multiplier $f_{V}$ in Eq. (18), with that the firm value is related to the cash flows, and the target debt ratio $\frac{D}{V}$ of the respective firms, we can also determine $f_{D}=\frac{D}{V}\cdot f_{V}$ and $f_{E}=(1-\frac{D}{V})\cdot f_{V}$. Therefore, we can write for $k_{E}$:

$$\begin{aligned}\displaystyle k_{E}=\frac{\left(1-p\right)\cdot\left(u+u\cdot f_{E}+\left(u-1\right)\cdot f_{D}-f_{D}\cdot c\cdot\left(1-\tau\right)\right)}{f_{E}}-1\end{aligned}$$

We simultaneously adjust both the factors $d$ and $k_{U}$ until the endogenous interest rate $c$ and the endogenous cost of equity $k_{E}$ match the empirically observed interest rate $\hat{c}$ and cost of equity $\hat{k}_{E}$. We perform this two-condition-approach for different levels of bankruptcy costs relative to the maximum value $\hat{\alpha}$ of the respective firms. As a final step, capital cost $k_{V}$ of the levered firms is calculated using Eq. (17).

In Table 4 we present the results of the calibration of Range Resources for different levels of bankruptcy costs $\alpha$. The maximum bankruptcy costs $\hat{\alpha}$, which can be calculated from the given parameters $\hat{k}_{E}$ and $\hat{c}$, are 61.0%. Since the cost of equity $k_{E}$ and the interest rate $c$ within the scope of this calibration have the same level for different bankruptcy costs $\alpha$, $k_{V}$ with a value of 5.6% is also the same throughout. The higher the bankruptcy costs $\alpha$, the lower $k_{U}$ must be chosen to ensure that $\hat{k}_{E}$ is equal to $k_{E}$. Likewise applies to the factor $d$ that this also rises with rising bankruptcy costs, in order to ensure $\hat{c}$ equals $c$ due to the calibration.

In general, it can be seen that the differences between $k_{V}$ and $k_{U}$ increase significantly with rising bankruptcy costs $\alpha$. This is also reflected in increasing pricing errors PE. With bankruptcy costs of 0%, the pricing error PE with 0.3% is economically negligible. However, even if bankruptcy costs of 40% are taken into account, equating $k_{U}$ and $k_{V}$ would lead to a pricing error of 58%. With the maximum bankruptcy costs of 61.0%, the pricing error strongly rises above 200%.

**Table 4:** Results from the calibration of Range Resources

$\alpha$	0%	10%	20%	30%	40%	50%	55%	$\hat{\alpha}$
$k_{U}$	5.6%	5.1%	4.7%	4.2%	3.8%	3.4%	3.2%	2.9%
$k_{V}$	5.6%	5.6%	5.6%	5.6%	5.6%	5.6%	5.6%	5.6%
$f_{V}$	17.3%	17.3%	17.3%	17.3%	17.3%	17.3%	17.3%	17.3%
$d$	0.41	0.51	0.60	0.70	0.79	0.89	0.93	0.99
$k_{E}$	7.6%	7.6%	7.6%	7.6%	7.6%	7.6%	7.6%	7.6%
$k_{D}$	4.1%	4.1%	4.1%	4.1%	4.1%	4.1%	4.1%	4.1%
$g$	–1.3%	–0.8%	–0.2%	0.3%	0.8%	1.3%	1.5%	1.8%
$k_{V}-k_{U}$	0.0%	0.5%	0.9%	1.3%	1.8%	2.2%	2.4%	2.6%
*PE*	0.3%	8.0%	18.5%	33.8%	58.1%	103.1%	143.8%	237.8%
$DtS$	29.3%	29.3%	29.3%	29.3%	29.3%	29.3%	29.3%	29.3%

As described in assumption 9, at any point in time, the residual value $d\cdot X_{t}+d\cdot V_{t}-\alpha\cdot V_{t}$ in the down state must be less than interest and repayments $c\cdot D_{t}+D_{t}$ to satisfy the default condition. For this reason, the following inequality must be met:

22 $$\begin{aligned}d\cdot X_{t}+d\cdot V_{t}-\alpha\cdot V_{t}<c\cdot D_{t}+D_{t}\end{aligned}$$

Dividing inequality (22) by $V_{t}$ gives the following simplification:

23 $$\begin{aligned}\frac{d}{f_{V}}+d-\alpha<(1+c)\cdot\frac{D}{V}\end{aligned}$$

In case of Range Resources Corp., we illustrate the validity of this inequality by introducing the parameter Distance to Solvency (*DtS*). Thereby, we set the residual value $d\cdot X_{t}+d\cdot V_{t}-\alpha\cdot V_{t}$ in proportion to the value of interest and repayments $c\cdot D_{t}+D_{t}$. Table 4 demonstrates that in the case of Range Resources Corp., the residual value is 29.3% lower than the required value for repayment and interest to achieve solvency of the firm.

In order to show that these results are not only valid for firms with a severe probability of default, we calibrate the present model using large German stock corporations. The general scope of this study comprises 130 companies from the German DAX, MDAX and SDAX indices, of which we can finally use the data of 29 companies to conduct the calibration of our model as of January 1, 2018. However, we do not expect any major fluctuations in results depending on the valuation date concerning the historical input data. We obtain key corporate data, such as market capitalization, balance sheet information and rating grades from the standard information data service Thompson Reuters Eikon, subtracted on March 14, 2019. Furthermore, we use publicly available information from the Deutsche Bundesbank for interest rates as well as the rating agencies Moody’s, Standard Poors and Fitch.

We first take the risk-free interest rate $r_{f}$ from the implicit spot rate with a maturity equal to the time to maturity of that German sovereign bond with a maximum lifetime of 30 years. The necessary parameters for this are provided by Deutsche Bundesbank, calculated using the approach by Svensson (1994) and result in a value of 1.29%. The debt ratio $\frac{D}{V}$ results from historical balance sheet data for the years 2014 to 2017. From this, we form the arithmetic mean as a proxy for the target debt ratio of the firms (see Appendix IV, Table 7). Additionally, for the conditional growth rate of cash flow and firm value $u$ in case of surivorship we use the target inflation rate of the European Central Bank of 2% per period. As a result, we fix the value of the factor $u$ at 1.02 for all firms. For the corporate tax rate $\tau$, we choose a fixed tax rate of 30%, since this tax rate is close to the average actual tax burden from corporate tax in the German tax system. Moreover, all firms are supposed to be subject to similar tax rates in the long run. In line with the procedure above external rating information available from the well-known rating agencies Moody’s, SP, and Fitch at the time of valuation. Among our basic scope of 130 companies, 45 companies have an external rating. Since all rating agencies publish historical default frequencies to indicate the rating scores, we use these percentages as input for the derivation of a default probability in our model. The respective rating grades and corresponding probabilities of default are listed in Table 8 in Appendix IV. Moreover, we obtain the interest rate $c$ from the bond yields of the respective debt capital bonds issued. If a company has issued several bonds, the spreads are weighted by volume and maturity. As we find bonds issued by 29 of the 45 companies which have an external rating, we use these 29 firms as final scope of our study. The empirically observed values for $\hat{c}$ are listed in Table 7 in Appendix IV.

Table 5 shows the difference between the cost of capital of the levered companies $k_{V}$ and the unlevered companies $k_{U}$ depending on the respective level of $\alpha$. In column 2, we represent the maximum bankruptcy costs $\hat{\alpha}$ of the respective firms of which we present different levels in columns 3 to 7, ranging from no bankruptcy costs $0\cdot\hat{\alpha}$ to maximum bankruptcy costs $\hat{\alpha}$.

All companies within the scope of this study also confirm the results found at Range Resources Corp: The difference between $k_{V}$ and $k_{U}$ increases with rising bankruptcy costs $\alpha$. Without bankruptcy costs, as shown in column 3, the deviations are marginal but still observable.^7^ However, if we consider relatively low bankruptcy costs of $\frac{1}{4}\cdot\hat{\alpha}$, we already see remarkable deviations in cost of capital of 0.95% on average with a maximum of 1.57% for Infineon. In case of the maximum bankruptcy costs at $\hat{\alpha}$, we notice that the deviations increase on average to 3.67%, with a maximum value of 6.03%. The deviations become even more conspicuous when we consider the pricing error in relation to the firm value. In Table 6, we represent the pricing error analogous to the approach of Eq. (21). The higher the percentage value of the pricing error, the higher the company would be valued if $k_{U}$ was used rather than $k_{V}$. With bankruptcy costs of 0%, the pricing error is also marginal. However, if a quarter of the maximum bankruptcy costs affects the companies, we can already notice a pricing error of 32.1% on average. At 50% of the maximum bankruptcy costs, companies are remarkably overpriced by an average of 95.5%. At 75% of the maximum bankruptcy costs, the mispricing even ranges between 104.9 and 309.5%. With maximum bankruptcy costs $\hat{\alpha}$, the pricing error reveals tremendous values.

**Table 5:** Difference $k_{V}$ and $k_{U}$. *The table illustrates the difference between the cost of capital *$k_{V}$* of a levered firm and the cost of capital *$k_{U}$* of an unlevered firm depending on the respective level of maximum bankruptcy costs *$\hat{\alpha}$

Difference $k_{V}-k_{U}$						
Company	$\hat{\alpha}$	$0\cdot\hat{\alpha}$	$0.25\cdot\hat{\alpha}$	$0.5\cdot\hat{\alpha}$	$0.75\cdot\hat{\alpha}$	$\hat{\alpha}$
BASF	65.2%	0.00%	1.19%	2.35%	3.47%	4.60%
Bayer	60.6%	0.01%	1.19%	2.34%	3.47%	4.60%
BMW	32.4%	0.02%	0.70%	1.37%	2.04%	2.71%
Continental	73.8%	0.00%	1.45%	2.86%	4.24%	5.55%
Covestro	56.9%	0.01%	1.12%	2.20%	3.27%	4.31%
Daimler	34.3%	0.02%	0.73%	1.42%	2.11%	2.78%
Deutsche Börse	17.5%	0.02%	0.27%	0.53%	0.79%	1.05%
Deutsche Lufthansa	47.3%	0.01%	0.71%	1.40%	2.08%	2.77%
Deutsche Post	64.1%	0.00%	1.05%	2.08%	3.09%	4.07%
Deutsche Telekom	40.4%	0.01%	0.71%	1.38%	2.06%	2.74%
Fresenius	54.1%	0.01%	0.87%	1.71%	2.54%	3.37%
Fresenius Med.Care	58.2%	0.01%	0.91%	1.79%	2.67%	3.49%
Heidelbergcement	65.4%	0.01%	1.24%	2.44%	3.62%	4.73%
Henkel	78.6%	0.00%	1.11%	2.19%	3.26%	4.30%
Infineon	79.4%	0.00%	1.57%	3.09%	4.57%	6.03%
Merck	54.5%	0.01%	0.87%	1.71%	2.54%	3.38%
RWE	49.5%	0.02%	0.94%	1.85%	2.73%	3.61%
SAP	68.7%	0.00%	1.06%	2.10%	3.11%	4.12%
Siemens	54.3%	0.01%	0.93%	1.85%	2.73%	3.64%
Thyssenkrupp	35.2%	0.03%	0.77%	1.50%	2.22%	2.91%
Volkswagen	36.3%	0.02%	0.83%	1.62%	2.41%	3.18%
Evonik Industries	72.3%	0.00%	1.06%	2.09%	3.11%	4.12%
Hochtief	49.1%	0.01%	0.71%	1.40%	2.08%	2.75%
Lanxess	57.1%	0.01%	1.12%	2.21%	3.29%	4.29%
MTU Aero Engines	55.0%	0.01%	0.80%	1.57%	2.34%	3.11%
Schaeffler	41.7%	0.03%	0.87%	1.69%	2.50%	3.31%
Bilfinger Berger	75.7%	0.00%	1.25%	2.47%	3.66%	4.78%
Heidelb. Druck.	62.2%	0.02%	1.03%	2.02%	2.97%	3.93%
Hornbach	77.5%	0.00%	0.59%	1.16%	1.72%	2.20%
Mean	55.8%	0.01%	0.95%	1.88%	2.78%	3.67%

**Table 6:** Pricing Error. *The table illustrates the pricing error of the firm value depending on the respective level of maximum bankruptcy costs *$\hat{\alpha}$

Pricing Error [PE]						
Company	$\hat{\alpha}$	$0\cdot\hat{\alpha}$	$0.25\cdot\hat{\alpha}$	$0.5\cdot\hat{\alpha}$	$0.75\cdot\hat{\alpha}$	$\hat{\alpha}$
BASF	65.2%	0.1%	34.4%	103.0%	309.5%	259 986.8%
Bayer	60.6%	0.1%	34.0%	101.7%	303.9%	43 865.7%
BMW	32.4%	0.7%	34.5%	101.8%	302.2%	22 040.2%
Continental	73.8%	0.1%	34.1%	102.2%	305.6%	167 677.9%
Covestro	56.9%	0.2%	32.9%	98.3%	293.7%	96 549.3%
Daimler	34.3%	0.7%	34.4%	101.8%	302.2%	56 145.2%
Deutsche Börse	17.5%	1.6%	35.3%	101.5%	299.1%	11 245.9%
Deutsche Lufthansa	47.3%	0.3%	31.4%	93.5%	278.5%	27 492.5%
Deutsche Post	64.1%	0.1%	33.9%	101.5%	304.1%	413 349.6%
Deutsche Telekom	40.4%	0.5%	33.3%	98.9%	295.6%	69 506.4%
Fresenius	54.1%	0.2%	31.5%	94.0%	280.9%	39 857.3%
Fresenius Med.Care	58.2%	0.2%	31.1%	92.8%	276.8%	432 198.9%
Heidelbergcement	65.4%	0.1%	32.4%	96.7%	289.1%	480 734.9%
Henkel	78.6%	0.0%	33.8%	101.4%	304.1%	691 764.8%
Infineon	79.4%	0.0%	33.8%	101.3%	303.5%	141 193.4%
Merck	54.5%	0.2%	33.0%	98.8%	295.9%	65 215.0%
RWE	49.5%	0.6%	33.2%	98.4%	293.8%	94 441.6%
SAP	68.7%	0.0%	33.9%	101.6%	304.6%	62 562.6%
Siemens	54.3%	0.2%	34.1%	101.8%	305.7%	73 183.9%
Thyssenkrupp	35.2%	0.9%	30.2%	88.6%	263.2%	92 847.0%
Volkswagen	36.3%	0.6%	34.3%	101.5%	302.0%	40 325.8%
Evonik Industries	72.3%	0.0%	32.6%	97.5%	291.4%	57 393.0%
Hochtief	49.1%	0.3%	32.4%	96.6%	288.0%	46 056.1%
Lanxess	57.1%	0.2%	32.5%	97.0%	289.1%	117 277.4%
MTU Aero Engines	55.0%	0.2%	32.3%	96.4%	287.3%	32 843.2%
Schaeffler	41.7%	1.0%	33.3%	98.0%	292.4%	125 695.2%
Bilfinger Berger	75.7%	0.0%	30.3%	90.6%	270.5%	168 501.9%
Heidelb. Druck.	62.2%	0.4%	26.2%	77.3%	229.9%	48 245.9%
Hornbach	77.5%	0.1%	12.0%	35.4%	104.9%	80 193.9%
Mean	55.8%	0.3%	32.1%	95.5%	285.1%	139 944.5%

## Conclusion

The practical treatment that the company cost of capital does not change (approximately) when the firm follows a one-time, substantial change in the debt ratio is not an analytical relationship but a simplification. Numerical inspections justify equating the cost $k_{V}$ of a levered company and the cost $k_{U}$ of an unlevered one due to extremely small deviations in case the company has no default risk.

However, in the case of default risk and with bankruptcy costs, the cost of capital $k_{V}$ of a levered company is significantly higher than the cost of capital $k_{U}$ of an identical unlevered firm. In this context, $k_{V}> k_{U}$ applies to any level of bankruptcy costs and can lead to significant pricing errors. Calibrating the cost of capital of real listed firms within the presented DCF model framework confirms, on the one hand, the general applicability and, on the other hand, the high pricing errors even for firms with relatively good rating grades. Especially the level of bankruptcy costs significantly affects the difference between $k_{V}$ and $k_{U}$. Accordingly, in the event of bankruptcy in which no residual value of the firm remains, the deviations between $k_{V}$ and $k_{U}$ amount to 3.67 percentage points on average with serious pricing errors.

The consideration of bankruptcy costs and default risk provides specific challenges in practical applications. Since any given firm usually has no observable bankruptcy costs – while it is alive – a reference to experiences of historical default must be applied. However, empirical studies concerning direct and indirect bankruptcy costs exhibit highly heterogeneous results making it necessary to prudently treat this variable.

A further main driver of the difference between $k_{V}$ and $k_{U}$ is the risk premium of $k_{U}$ relative to the risk-free rate $r_{f}$. In the present case, the observed cost of equity $\hat{k_{E}}$ was used to indirectly determine $k_{U}$. Again, the estimates are primarily sensitive in terms of the magnitude of the market risk premium and to a minor extent to the correct beta factor of the particular firm.

In general, the results of this paper force us to rethink the established valuation approaches. Once default risk plays a noticeable role and branches with high bankruptcy costs are considered, the company cost of capital *does* significantly depend on the debt ratio and this relationship needs to be taken into account for accurate valuation purposes. Therefore, this effect should be part of all other, potentially even more sophisticated company valuation models when default risk matters and a change in the debt ratio is intended.

## Appendix

### Appendix I: Determination of replicating portfolio of firm value in Leland model

In order to determine the replicating portfolio $RP$ for the firm value $V\left(U\right)$, we follow the typical valuation in a Black-Scholes world. According to delta hedging, the replicating portfolio needs to satisfy two conditions: (1) the value matching condition, and (2) the delta matching condition, i.e., the first derivative of the claim value $V\left(U\right)$ for the state variable $U$ needs to coincide with that of the replicating portfolio. In principle, the replicating portfolio only consists out of units $W_{U}$ of the unlevered firm $U$ and a holding $W_{f}$ (measured in monetary units) in the riskfree asset:

$$RP=W_{U}\cdot U+W_{f}.$$

As a result of the delta heding condition (2), it must hold for $W_{U}$:

$$\begin{aligned}\displaystyle&\displaystyle\frac{\partial RP}{\partial U}=\frac{\partial V\left(U\right)}{\partial U}\\ \displaystyle&\displaystyle W_{U}=1+\frac{a\cdot U_{B}+\frac{c\cdot\tau}{r}}{U\cdot\frac{\sigma^{2}}{2r}}\left(\frac{U}{U_{B}}\right)^{-\frac{2r}{\sigma^{2}}}\end{aligned}$$

Hence, the value matching condition (1) results in riskfree holdings $W_{f}$:

$$\begin{aligned}\displaystyle W_{f}&\displaystyle=V\left(U\right)-W_{U}\cdot U\\ \displaystyle&\displaystyle=V\left(U\right)-U-\frac{a\cdot U_{B}+\frac{c\cdot\tau}{r}}{\frac{\sigma^{2}}{2r}}\left(\frac{U}{U_{B}}\right)^{-\frac{2r}{\sigma^{2}}}\\ \displaystyle&\displaystyle=\frac{c\cdot\tau}{r}+\left(\frac{U}{U_{B}}\right)^{-\frac{2r}{\sigma^{2}}}\cdot\left(1+\frac{2r}{\sigma^{2}}\right)\cdot\left(-a\cdot U_{B}\cdot-\frac{c\cdot\tau}{r}\right)\end{aligned}$$

Ultimately, the required relative portfolio weights $w_{U}$ and $w_{f}$, respectively, follow from

$$\begin{aligned}\displaystyle&\displaystyle w_{U}=\frac{W_{U}\cdot U}{RP}=\frac{U+\frac{a\cdot U_{B}+\frac{c\cdot\tau}{r}}{\frac{\sigma^{2}}{2r}}\left(\frac{U}{U_{B}}\right)^{-\frac{2r}{\sigma^{2}}}}{V\left(U\right)},\\ \displaystyle&\displaystyle w_{f}=\frac{W_{f}}{RP}=1-w_{U}.\end{aligned}$$

### Appendix II: Proof that the value of the levered firm $V_{t}$ is homogenous of degree one in $X_{t}$ and that the debt level dependent on the cash flow level leads to a constant debt ratio $\frac{D}{V}$

To have a constant debt ratio $\frac{D}{V}$ over time, we formally show that the following three properties hold within the model framework we have selected:

#### *Property 1 *

The debt volume $D_{t}$ is in a proportional relationship with $X_{t}$ by factor $f_{D}$.

#### *Property 2 *

The interest rate $c$ is constant and independent of $X_{t}$.

#### *Property 3 *

The levered firm value $V_{t}$ is homogenous of the first degree in $X_{t}$.

While property 1 holds by assumption, we show the validity of properties 2 and 3 with the following implications. Since the implication in both directions holds, a firm satisfying the three properties must always exist.

#### Properties 1 and 3 imply property 2

The interest rate $c$ endogenously follows from Eq. (10). Given that properties 1 and 3 apply, $c$ is apparently constant with:

$$\begin{aligned}\displaystyle c=\frac{D_{t}\cdot(1+r_{f})+q\cdot D_{t}-D_{t}-q\cdot\left(d\cdot X_{t}+\left(d-\alpha\right)\cdot V_{t}\right)}{D_{t}-q\cdot D_{t}}\end{aligned}$$

#### Properties 1 and 2 imply property 3

If properties 1 and 2 apply, $V_{t}$ is homogenous of first degree in $X_{t}$. This is because all future revenues obtained by the firm are proportionate to $X_{t}$. If $X_{t}$ at time $t$ increases by an arbitrary factor $m$, the cash flows $X_{t+s}$ at a later date $t+s$ will also increase by factor $m$ due to the assumed stochastic process of $X_{t}$. Likewise, the tax shields $D_{t+s-1}\cdot c\cdot\tau$ obtained at time $t+s$ equal $X_{t+s-1}\cdot f_{D}\cdot c\cdot\tau$ are also altered by factor $m$ when the current cash flow $X_{t}$ is multiplied by $m$. As a result of constant discount rates $k_{U}$ and $r_{f}$, the current firm value $V_{t}$ increases by factor $m$ when all future cash flows rise by $m$. Hence, we have a proportionate relationship between the firm value $V_{t}$ and the current cash flows $X_{t}$:

$$V_{t}=f_{V}\cdot X_{t}$$

**Conclusion.** Since property 1 is always valid due to assumption 11 and properties 2 and 3 are mutually explanatory, we can conclude that there exists a company for which the three properties are satisfied.

Furthermore, as both multipliers $f_{D}$ and $f_{V}$ are constants, we can confirm that the debt ratio $\frac{D}{V}$ is also constant with:

$$\frac{D}{V}=\frac{f_{D}\cdot X_{t}}{f_{V}\cdot X_{t}}=\frac{f_{D}}{f_{V}}$$

### Appendix III: Proof of the Property that Cost $k_{V}$ of a Levered Firm exceeds the Cost $k_{U}$ of an Unlevered Firm

We show that the general relationship $k_{V}> k_{U}$ for the framework in Sect. 3 holds – even in the absence of bankruptcy costs $\alpha$. We illustrate this by replicating the levered firm $V_{t}$ by a portfolio consisting of a risk-free bond and the otherwise identical but unlevered firm $U_{t}$; i.e., the (expected) total revenues of the replicating portfolio at the subsequent date $t+1$ coincide with those of the firm value $V_{t+1}$ in the corresponding state.

For this purpose, we first refer to the value of an unlevered firm $U_{t}$ that is supposed to attain two different states with state-dependent conditionally expected total value at time $t+1$ equal to $U^{\text{up}}$ and $U^{\text{down}}$, respectively.

Since the risk-free bond is redeemed at time $t+1$ regardless of the respective state including the risk-free interest rate $r_{f}$, we have:

Regarding the levered firm, there are two relevant states at time $t+1$. The payoffs in these states differ from those of the states of the unlevered firm by the tax shield $D_{t}\cdot c\cdot\tau$ in the up-state and bankruptcy costs $\alpha\cdot V_{t}$ in the down-state. Moreover the firm value is $u\cdot V_{t}$ or $d\cdot V_{t}$ for the levered firm rather than $u\cdot U_{t}$ and $d\cdot U_{t}$ for the unlevered company.

In order to determine the value of the firm $V_{t}$, we demonstrate that we can replicate its expected payoffs in both states using a portfolio of the unlevered firm $U_{t}$ and the risk-free bond. For this purpose, $n_{U}$ denotes the number of units in firm $U_{t}$ and $n_{f}$ the number of the initial investment in the risk-free bond. Thus, we can represent both the total expectation in the up-state $V^{\text{up}}$ and down-state $V^{\text{down}}$ for the company $V_{t+1}$ analogously to an investment in the unlevered firm $U_{t}$ and the risk-free bond:

Accordingly, we obtain the following equations for both states:

$$\begin{aligned}\displaystyle u\cdot X_{t}\cdot\left(1+f_{V}\right)+c\cdot\tau\cdot D_{t}=n_{U}\cdot\left(u\cdot X_{t}\cdot\left(1+f_{U}\right)\right)+n_{f}\cdot\left(1+r_{f}\right)\\ \displaystyle d\cdot X_{t}\cdot\left(1+f_{V}\right)-\alpha\cdot V_{t}=n_{U}\cdot\left(d\cdot X_{t}\cdot\left(1+f_{U}\right)\right)+n_{f}\cdot\left(1+r_{f}\right)\end{aligned}$$

The solution for these two equations for the two portfolio holdings $n_{U}$ and $n_{f}$ amounts to:

24 $$\begin{aligned}n_{U}=\frac{V^{\text{up}}-V^{\text{down}}}{U^{\text{up}}-U^{\text{down}}}=\frac{\left(1+f_{V}\right)\cdot\left(u-d\right)\cdot X_{t}+\tau\cdot c\cdot D_{t}+\alpha\cdot V_{t}}{\left(1+f_{U}\right)\cdot\left(u-d\right)\cdot X_{t}}\end{aligned}$$

25 $$\begin{aligned}n_{f}=\frac{V^{\text{down}}-U^{\text{down}}\cdot n_{U}}{1+r_{f}}=\frac{d\cdot X_{t}\cdot\left(1+f_{V}\right)-\alpha\cdot V_{t}-n_{U}\cdot d\cdot X_{t}\cdot\left(1+f_{U}\right)}{1+r_{f}}\end{aligned}$$

To verify that the risk of the levered firm and thus the cost of capital $k_{V}$ is larger than $k_{U}$ we only need to demonstrate that there is a short position in the risk-free bond, $n_{f}<0$. This is because of the well-known leverage effect. Using the replicating portfolio for $V_{t}$, we can write the cost of capital $k_{V}$ as a weighted expected return of its components:

$$k_{V}=\frac{n_{U}\cdot U_{t}}{V_{t}}\cdot k_{U}+\frac{n_{f}}{V_{t}}\cdot r_{f},$$

with

$$\frac{n_{U}\cdot U_{t}}{V_{t}}+\frac{n_{f}}{V_{t}}=1.$$

From the assumption $k_{U}> r_{f}$, we obtain $k_{V}> k_{U}$ if and only if $n_{f}$ is negative. Using Eq. (25), we can recognize that a negative holding $n_{f}$ is associated with a negative numerator of the fraction:

26 $$\begin{aligned}d\cdot X_{t}\cdot\left(1+f_{V}\right)-\alpha\cdot V_{t}+n_{U}\cdot d\cdot X_{t}\cdot\left(1+f_{U}\right)<0\end{aligned}$$

If we solve Eq. (26) for $n_{U}$, we obtain:

$$n_{U}> \frac{1+f_{V}}{1+f_{U}}-\frac{\alpha\cdot V_{t}}{d\cdot X_{t}\cdot\left(1+f_{U}\right)}$$

Since $n_{U}> \frac{1+f_{V}}{1+f_{U}}$ always holds due to Eq. (24), we can directly conclude that Eq. (26) is always valid.

### Appendix IV: Details from the Model Calibration

**Table 7:** Target Debt Ratios. *The table illustrates the target debt ratio resulting from average historical balance sheet data for the years 2014 to 2017, the cost of equity *$\hat{k}_{E}$*, the equity beta factor *$\beta_{E}$* over three years and the debt interest rate *$\hat{c}$* of all firms within the final scope*

Company	$\frac{D}{V}$	$\beta_{E}$	$\hat{k}_{E}$	$\hat{c}$
BASF	34.0%	0.98	8.88%	2.09%
Bayer	38.7%	1.06	9.49%	1.94%
BMW	66.1%	1.16	10.31%	1.80%
Continental	27.1%	1.06	9.50%	2.33%
Covestro	43.2%	1.06	9.54%	2.01%
Daimler	65.9%	1.13	10.08%	2.04%
Deutsche Börse	77.4%	0.81	7.53%	1.73%
Deutsche Lufthansa	50.6%	0.80	7.50%	2.01%
Deutsche Post	33.1%	0.88	8.11%	1.92%
Deutsche Telekom	59.8%	0.93	8.53%	2.16%
Fresenius	45.9%	0.86	7.97%	2.24%
Fresenius Med.Care	42.8%	0.84	7.77%	2.44%
Heidelbergcement	35.9%	1.02	9.21%	2.31%
Henkel	15.1%	0.75	7.12%	1.57%
Infineon	20.7%	1.06	9.53%	1.97%
Merck	43.3%	0.85	7.89%	2.00%
RWE	61.8%	1.01	9.14%	3.50%
SAP	26.8%	0.83	7.69%	1.76%
Siemens	43.7%	0.92	8.45%	1.95%
Thyssenkrupp	70.8%	1.18	10.41%	2.74%
Volkswagen	64.3%	1.23	10.84%	2.02%
Evonik Industries	23.7%	0.78	7.35%	1.93%
Hochtief	50.5%	0.77	7.24%	2.29%
Lanxess	44.4%	1.06	9.53%	2.26%
MTU Aero Engines	45.8%	0.77	7.27%	2.50%
Schaeffler	73.5%	1.10	9.82%	3.78%
Bilfinger Berger	25.4%	0.89	8.17%	2.60%
Heidelb. Druck.	63.4%	0.88	8.09%	5.51%
Hornbach	35.4%	0.41	4.46%	5.00%

**Table 8:** Ratings and Probabilities of Default. *The table illustrates the ratings of StandardPoors, Moody’s and Fitch and the corresponding 10-year probabilities of default. If ratings from more than one agency are available, we calculate the arithmetic mean and convert the 10-year-PD into an average 1‑year-PD*

Company	SP	Moody’s	Fitch	10-Y-PD SP	10-Y-PD Moody’s	10-Y-PD Fitch	Average 1‑Y-PD
BASF	A	A1	A+	1.41%	1.42%	0.70%	0.12%
Bayer	A$-$			2.19%			0.22%
BMW		A1			1.42%		0.14%
Continental	BBB$+$		BBB$+$	2.98%		2.03%	0.25%
Covestro		Baa1			4.89%		0.50%
Daimler	A	A2	A$-$	1.41%	1.42%	2.28%	0.17%
Deutsche Börse	AA			0.77%			0.08%
Deutsche Lufthansa	BBB$-$	Baa3		6.95%	4.89%		0.61%
Deutsche Post		A3	BBB$+$		1.42%	2.03%	0.17%
Deutsche Telekom	BBB$+$	Baa1	BBB$+$	2.98%	4.89%	2.03%	0.33%
Fresenius	BBB$-$	Baa3	BBB$-$	6.95%	4.89%	7.21%	0.65%
Fresenius Med.Care			BBB$-$			7.21%	0.75%
Heidelbergcement	BBB$-$	Baa3	BBB$-$	6.95%	4.89%	7.21%	0.65%
Henkel	A	A2	A	1.41%	1.42%	2.04%	0.16%
Infineon	BBB			3.76%			0.38%
Merck	A	Baa3		1.41%	4.89%		0.32%
RWE	BBB$-$		A$-$	6.95%		2.28%	0.47%
SAP	A			1.41%			0.14%
Siemens		A1			1.42%		0.14%
Thyssenkrupp	BB	Ba2	BB$+$	13.33%	19.86%	9.92%	1.54%
Volkswagen	BBB$+$	A3		2.98%	1.42%		0.22%
Evonik Industries	BBB$+$	Baa1		2.98%	4.89%		0.40%
Hochtief	BBB			3.76%			0.38%
Lanxess	BBB$-$	Baa2		6.95%	4.89%		0.61%
MTU Aero Engines			BBB			4.07%	0.41%
Schaeffler		Baa3	BBB$-$		4.89%	7.21%	0.62%
Bilfinger Berger	BB$+$			10.14%			1.06%
Heidelb. Druck.	B$+$			21.40%			2.38%
Hornbach		B3			46.12%		6.00%

## Footnotes

1 The consideration of an unlevered firm first is consistent with the Leland mode from Sect. 2 where the value of a levered firm can be represented as the value of a replicating portfolio out of an unlevered firm and a risk-free asset. Thus, the unlevered firm value is needed to price the levered firm accordingly. An alternative approach is introduced by Baule (2019) who regards a CAPM-like market price of risk to value the levered firm rather than the value of an unlevered firm. Economically speaking, the valuation of a firm requires knowledge of the market price of risk irrespective of the fact whether it is contained in a CAPM-valuation formula or implicitly in the value of the unlevered firm.

2 In our model, a default is triggered whenever the down-state occurs. Since the reason for why a default takes place, i.e., overindebtedness versus illiquidity, is not relevant for our research question, we follow the notion of a reduced-form default risk modeling. Using a structural framework based on perfect markets such as that proposed by Kruschwitz et al. (2015), one can see that illiquidity and overindebtedness do not necessarily occur synchronously.

3 In case of a levered firm, we have the notion of an exogenous default that occurs if and only if the firm attains the down state. A company without debt capital but which is nevertheless exposed to default risk could undoubtedly also be represented in the existing model framework by integrating a payment obligation $D> 0$ in Sect. 3.2 with an interest rate $c=0$ and a positive exogenous probability of default.

4 Arzac and Glosten (2005) already showed in their Eq. (17) that the value of the unlevered firm $U_{t}$ under perpetual growth can be represented as a constant multiple of the free cash flow $X_{t}$. Kruschwitz and Löffler (2006) proved this relationship under more general conditions in their Appendix A.1.

5 In contrast to Leland (1994), who assumes a constant debt level, we deliberately deviate from this and switch to a market value-oriented view. We consider this financing policy to be more appropriate in a discrete model framework with regard to real firms.

6 Standard and Poors (2019), Global Corporate Average Default Rates (1981–2018).

7 In the case of the current 29 firms, we can also confirm the existence of inequality (23) and a positive value for the distance to solvency ($DtS$) according to assumption 9.
